# A case of conservatively managed gastric perforation at a recurrent hiatal hernia site after laparoscopic paraesophageal repair

**DOI:** 10.1016/j.ijscr.2023.108266

**Published:** 2023-04-26

**Authors:** Taichi Ogo, Yu Nishiyama, Kei Ishihara, Keiji Tsukahara, Mikito Inokuchi

**Affiliations:** Department of Surgery, Musashino Red Cross Hospital, Japan

**Keywords:** Conservative therapy, Paraesophageal hernia, Gastric perforation, Hiatal hernia, Case report

## Abstract

**Introduction and importance:**

Gastric perforation due to a hiatal hernia is a rare cause of acute abdominal pain that often requires surgical intervention. Conservative management for this condition is an effective option in certain cases, although fewer reports of this exist. Herein, we report a unique case of gastric perforation caused by a recurrent hiatal hernia that was successfully treated with conservative management.

**Case presentation:**

A 74-year-old man developed a high fever and an elevated inflammatory response on the third day after a laparoscopic paraesophageal hernia repair using a mesh. Computed tomography confirmed the recurrence of the hiatal hernia, with gastric fundal prolapse into the mediastinum and surgical emphysema in the gastric wall. This was followed by a gastric perforation within the mediastinum. The patient was treated using an ileus tube through the perforation site.

**Clinical discussion:**

In similar cases, if the clinical symptoms are mild, there are no signs of serious infection, and the perforation remains in the mediastinum and can be appropriately drained, conservative treatment is considered an option.

**Conclusion:**

Under favorable conditions, conservative management can be an option for gastric perforation in patients with recurrent hiatal hernias, which is a serious potential postoperative complication.

## Highlights

• Conservative therapy can be effective for gastric perforation in recurrent hiatal hernias
• Ileus tube has a good balance of flexibility and rigidity for mediastinal drainage
• Strong abdominal pressure should be avoided after giant hiatal hernia repair

## 1 Introduction

Gastric perforation owing to a hiatal hernia is a rare cause of acute abdominal pain. Paraesophageal hernias (PEH) represent 5–10 % of all hiatal hernias [1]. PEH is defined as the herniation of over one-third of the stomach into the mediastinum. Risk factors for developing PEH include conditions that lead to increased intra-abdominal pressure, such as chronic constipation, chronic cough, chronic obstructive airway disease, and heavy physical labor. The increase in intra-abdominal pressure creates a trans-diaphragmatic pressure gradient between the thorax and the abdominal cavity at the gastroesophageal junction [2]. Ultimately, the phrenoesophageal membrane becomes weakened and stretched, and the esophageal hiatus subsequently widens, resulting in progressive stomach herniation into the posterior mediastinum [2].

Gastric perforation owing to a hiatal hernia is associated with high morbidity and mortality, and emergency operative intervention is required in most cases. Bujoreanu et al. reported a high morbidity and mortality rate in the emergency repair of giant hiatal hernias. Approximately 35 % of patients developed pneumonia, 24.3 % developed end organ damage requiring intensive care, 5.4 % underwent revision surgery, and 5.4 % died within 30 days [3].

Previous studies, including a literature review by Meredith et al. in 1980, reported a mortality rate of up to 60 % in patients with hiatal hernias and gastric perforations [4]. They recommended wedge resection of perforated ulcers or vagotomy and pyloroplasty in patients with hyperacidity. Dieter et al. reported a lower mortality rate of 20 %, which may reflect a quicker diagnosis with modern imaging techniques, such as computed tomography (CT), improved resuscitation techniques, contemporary critical management, and novel antimicrobials [5]. They performed emergency surgery with wedge resection of the gastric perforation site and an omental patch. The hiatal hernia was not repaired.

Urgent evaluation and surgical management can be lifesaving in patients with poor health, such as sepsis or hemodynamic instability. In contrast, conservative management is an effective option for patients in good general health who have, for example, no signs of sepsis or hemodynamic instability. However, all six cases of gastric perforation within a hiatal hernia reported since 2001 underwent emergency laparotomy. To the best of our knowledge, there have been no reports of conservative management of gastric perforation within a hiatal hernia [4].

This report describes a unique case of gastric perforation caused by a recurrent hiatal hernia that was successfully treated with conservative management. The SCARE 2020 Guideline was used to increase robustness and transparency in reporting this surgical case [6].

## 2 Presentation of case

**Fig. 1:**
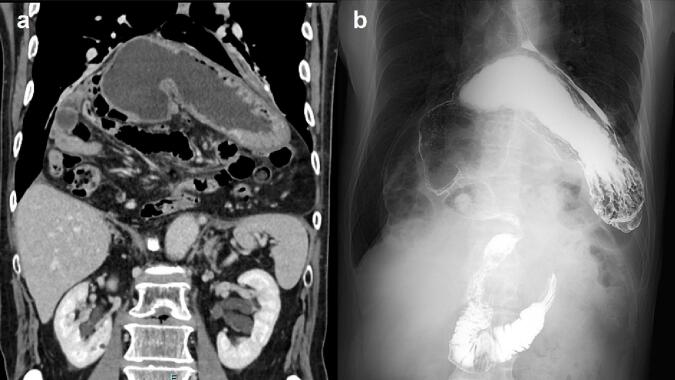
Coronal computed tomography showing (a) the intrathoracic stomach with organo-axial gastric volvulus and a large section of the small intestine. (b) Upper gastrointestinal series showing a volvulated stomach.

A 74-year-old man visited our hospital with gradually worsening left upper quadrant pain over a six-month period. The patient was using oral medication for hypertension and had a previous appendectomy. He was 180 cm tall and weighed 65 kg, with a body mass index (BMI) of 19.8 kg/m [2]. The findings of the physical examination were unremarkable. Chest and abdominal CT revealed an intrathoracic stomach with an organoaxial gastric volvulus involving a large section of the small intestine. Upper gastrointestinal series images also showed an upside-down stomach (Fig. 1). Based on these findings, we diagnosed type IV PEH.

The patient underwent laparoscopic hiatal hernia repair and a Toupet fundoplication under general anesthesia. Intraoperatively we found an organo-axial gastric volvulus with a small bowel prolapse of approximately 100 cm. Complete excision of the hernia sack was not possible, and we excised as much as possible. After the reduction of the stomach into the abdominal cavity, the length of the esophagus was about 3 cm, with a high degree of tension in the direction of the long axis. The esophageal hiatus was dilated to ≥6 cm and could not be closed sufficiently. To prevent a recurrence, we sutured the gastric wall and bilateral crura of the diaphragm, performed a mesh hiatoplasty using PARITEX™ HIATAL MESH fixed with a hernia stapler, and an anterior gastropexy.

**Fig. 2:**
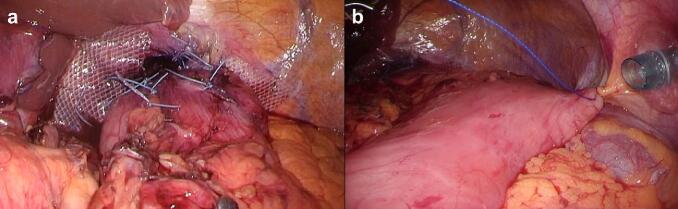
(a) Intra-operative findings after mesh hiatoplasty, (b) and anterior gastropexy.

On postoperative day 3, a high fever of 38.8 °C and elevated C-reactive protein (CRP), from 7.4 g mg/dL (postoperative day [POD] 1) to 34.0 mg/dL, were observed after the patient cleared his throat vigorously. CT revealed a recurrence of the hiatal hernia and intramural surgical emphysema of the prolapsed stomach (Fig. 2).

We considered whether a redo surgery was necessary but chose conservative treatment for the following three reasons: firstly, there was a sufficient risk of hernia recurrence even after reoperation; secondly, there was still a risk of delayed perforation after revision surgery; and thirdly, if the gastric wall was heavily damaged, a proximal gastrectomy may have been necessary. After explaining the above to the patient, conservative management was chosen.

**Fig. 3:**
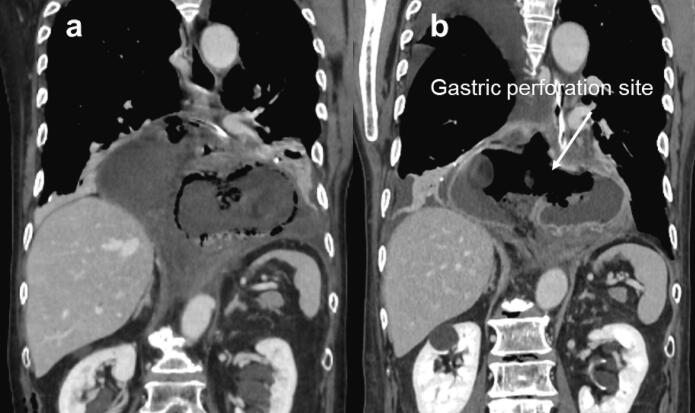
Computed tomography image showing (a) recurrence of the hiatal hernia and intramural surgical emphysema of the prolapsed stomach and (b) perforation of the gastric wall and free-air in the mediastinum.

**Fig. 4:**
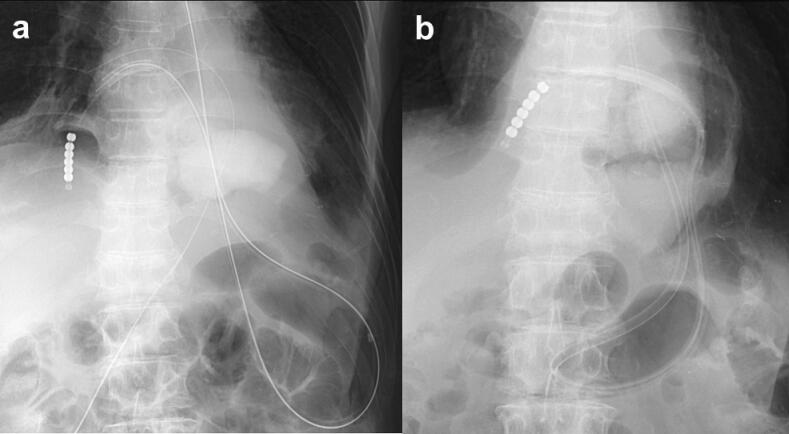
Drainage treatment initiated by (a) an ileus tube placed in the mediastinum through the perforation site. (b) The mediastinal cavity gradually decreased in size.

A nasogastric tube was placed for gastric decompression, and antibiotic therapy was initiated the same day. The white blood cell count (WBC) improved from 10,900 /μL (POD4) to 7700 /μL (POD11), and the CRP also improved from 39.4 mg/dL (POD4) to 15.5 mg/dL (POD11). However, a CT performed one week later revealed a 3 cm perforation on the right dorsal side of the wrapped gastric wall and free-air in the mediastinum (Fig. 3). Therefore, we placed a transnasal ileus tube into the mediastinum through the perforation guided by endoscopy and radiography and commenced drainage (Fig. 4a).

We could not insert the scope directly into the cavity because the perforation was in the right posterior wall of the stomach, which had been wrapped for the fundoplication. Therefore, we reversed the guidewire at the lower part of the gastric corpus, guided it through the perforation into the mediastinal cavity under fluoroscopic guidance, and subsequently inserted a long, soft, and resilient ileus tube. The ileus tube was connected to a vacuum system, and intermittent aspiration (50-s aspiration, 10-s pause) at −20 cmH2O was performed. The mediastinal cavity gradually decreased in size (Fig. 4b), and the deflection of the tube in the stomach was reduced so that the tip of the tube became shallower as the cavity shrank. Eventually, the tube tip fell into the stomach as the cavity closed.

The ileus tube was removed, and oral intake was initiated on postoperative day 27. The patient was on Total Parenteral Nutrition during this period. The patient was discharged from our hospital on postoperative day 39 and has been under outpatient observation for six months after surgery without any changes in his clinical condition. The recurrent hernia is under observation because his subjective symptoms have improved.

## 3 Discussion

Postoperative complications develop in 3–45 % of patients who undergo PEH repair [1]. The incidence of complications that requires reoperation within 30 days ranged from 1.6 to 4.9 %. Most of these cases are attributed to postoperative leakage, perioperative hernia recurrence, bleeding, obstruction of the fundoplication, or small bowel obstruction [7].

The current anatomical classification has evolved to include the categorization of hiatal hernias into Types I – IV. Type I hernias are sliding hiatal hernias, where the gastroesophageal junction migrates above the diaphragm. The stomach remains in its usual longitudinal alignment, and the fundus remains below the gastroesophageal junction. Type II hernias are pure paraesophageal hernias (PEH); the gastroesophageal junction remains in its normal position, but a portion of the fundus herniates through the esophageal hiatus adjacent to the esophagus. Type III hernias are a combination of Types I and II, with both the gastroesophageal junction and the fundus herniating through the hiatus, where the fundus lies above the gastroesophageal junction. Type IV hiatal hernias are characterized by the presence of a structure other than the stomach, such as the omentum, colon, or small bowel within the hernia sac [8].

Yano F et al. reported that the recurrence rate of Type III and IV hiatal hernia after laparoscopic fundoplication was 23 %, which decreased to 10 % with the addition of mesh reinforcement and gastropexy [9]. However, the vigorous clearing of his throat caused a substantial increase in intra-abdominal pressure in our patient, which caused the upper part of the stomach, including the fundoplication site, to prolapse back into the mediastinum, thereby damaging the stomach wall. The prolapsed stomach was likely constricted by the sutured esophageal hiatus, resulting in increased gastric pressure, blood flow disturbance, partial necrosis, and perforation of the damaged area.

The general condition of our patient was stable because gastric decompression resulted in only mild mediastinal contamination. Therefore, we decided on conservative management. In similar cases, if the clinical symptoms are mild, there are no signs of serious infection, and the perforation remains in the mediastinum and can be appropriately drained, conservative treatment is considered an option.

Conservative treatment options for delayed perforations include removable covered stents, endoscopic clips or sutures, and endoluminal vacuum therapy [8]. Stents are effective for esophageal perforation but ineffective for gastric perforation. Endoscopic closure of perforations with clips or sutures can successfully close esophageal and gastric perforations measuring <2 cm in size [10]. However, this approach is not indicated when the perforation is large, and the surrounding tissue is edematous and fragile, as in the present case. Endoluminal vacuum therapy has the benefit of providing source control with the use of continuous suction to clear bacterial contamination. In addition, it can be used to perform serial debridement of the leak cavity. Baylor University Medical Center reported 86 % success in healing perforations with endoluminal vacuum therapy [11]. But it has the disadvantage of the need for multiple endoscopies to exchange the endosponges [7].

In the present case, the giant hiatal hernia, defined as type III or IV, or when greater than half of the stomach is in the chest in the guidelines from SAGES 2013, was likely caused by frequent high abdominal pressure owing to the patient's vigorous clearing of his throat. Therefore, such etiologies must be considered in the postoperative management of hiatal hernia repair. It is important to teach patients how to expectorate and to use a nebulizer to help them expectorate. Furthermore, axial tension caused by the shortened esophagus should be released by performing an esophageal lengthening procedure, such as a Collis gastroplasty, to prevent a recurrence. Although more gentle surgical manipulation and postoperative management avoiding elevated abdominal pressures are necessary, conservative treatment with effective drainage is possible for gastric perforation, which is a serious complication. The ileus tube has a good balance of flexibility, which allows it to be inverted in the stomach, and rigidity, which prevents obstruction of the lumen. This balance was useful for conservative treatment in this case. The follow-up period was short, and since the number of cases of gastric perforation after PEH, which were treated conservatively, are few, we need more cases in order to have final conclusions.

## 4 Conclusion

Conservative therapy for gastric perforation in patients with recurrent hiatal hernias, which is a serious postoperative complication, can be an effective option in patients with good general health. We believe that this treatment option can be considered for future instances of similar cases.

## Consent for publication

Written informed consent was obtained from the patient for the publication of this case report and accompanying images. A copy of the written consent is available for review by the Editor-in-Chief of this journal on request.

## Ethical approval

Ethical approval is exempt/waived at our institution.

## Funding

This research did not receive any specific grant from funding agencies in the public, commercial, or not-for-profit sectors.

## CRediT authorship contribution statement

TO drafted the manuscript. YN, KI and KT participated in treating the patient. TO, KI and MI participated in the surgery. YN, KI, KT and MI were responsible for this paper. All of the authors read and approved the final manuscript.

## Guarantor

Taichi Ogo is the Guarantor who accept full responsibility for the work and the conduct of the study, had access to the data, and controlled the decision to publish.

## Declaration of competing interest

The authors have no competing interests to declare.
